# Physical activity counseling in primary care and family medicine residency training: a systematic review

**DOI:** 10.1186/s12909-018-1268-1

**Published:** 2018-07-03

**Authors:** Apichai Wattanapisit, Titiporn Tuangratananon, Sanhapan Thanamee

**Affiliations:** 1 School of Medicine, Walailak University, Thasala, Nakhon Si Thammarat, 80161 Thailand; 2 International Health Policy Program, Thailand, Ministry of Public Health, Muang, Nonthaburi, 11000 Thailand; 3 Thasala Hospital, Thasala, Nakhon Si Thammarat, 80160 Thailand

**Keywords:** Counseling, Education, Physical activity, Primary care, Residency training

## Abstract

**Background:**

Physical inactivity is a global public health challenge. Physical activity (PA) promotion in healthcare delivery systems is effective to reduce physical inactivity. A primary care setting provides an appropriate environment for PA counseling since it is a primary contact with primary care or family physicians encounter the majority of the population. Lack of knowledge and inadequate training in PA counseling is one of the most important barriers to PA promotion. The purpose of this systematic review was to evaluate PA counseling training in primary care residency programs.

**Methods:**

The authors systematically searched PubMed, Web of Science, Scopus and The Cochrane Library for articles published in English from 2000 to 2017. Articles regarding PA counseling in primary care residency training were extracted and outcomes assessed for this systematic review.

**Results:**

Based on the initial review, 378 articles were excluded (362 articles excluded based on titles and abstracts and 16 articles excluded based on full texts). Four articles were included in this review, addressed PA counseling curricula in primary care residency training. All studies included PA counseling training as part of obesity and healthy lifestyle training. The training improved knowledge among primary care residents, but may not necessarily result in better attitudes or self-efficacy, which could be improved by elective rotations that focus on improved attitudes, self-efficacy, and professional norms for PA counseling. Brief training in counseling did not improve quality nor increase the rate of counseling.

**Conclusions:**

This systematic review demonstrates a lack of evidence due to a small number of included studies. The heterogeneous outcomes from the minimal programs are needed to carefully interpret. However, this review sheds light on the importance of training in PA counseling in primary care residency programs. The development of training in PA counseling should focus on an approach that improves attitudes and the self-efficacy of primary care residents. Elective rotations, where residents voluntarily choose their subject, may provide the appropriate training period for PA counseling. Policymakers and academics should play an active role in the implementation of PA counseling as an essential competency for primary care physicians.

**Electronic supplementary material:**

The online version of this article (10.1186/s12909-018-1268-1) contains supplementary material, which is available to authorized users.

## Background

Physical inactivity is one of the most important public health issues of the 21^st^ century [1]. In 2016, approximately one quarter (23.3%) of adult population was physically inactive. Moreover, more than three quarters (76.3% of adolescents, 78.4% for boys and 84.4% for girls) of the younger, non-adult population was inactive [2]. According to the 2015 Global Burden of Disease Study, physical inactivity contributed to approximately 1.6 million deaths by increasing risk for several non-communicable diseases (NCDs) including; colon and rectum cancer, ischemic stroke, ischemic heart disease, and diabetes mellitus [3]. This “pandemic” of global physical inactivity contributes to a tremendous economic loss [4].

Physical activity (PA) promotion in healthcare delivery systems is an effective means by which to reduce physical inactivity and to ensure clinical benefit for patients [5]. Health-professional advice promotes regular PA and increases satisfaction with medical care [6]. Moreover, patients respect physicians and consider their advice credible and an excellent source for health-related information and guidance [7]. Encounters between physicians and patients are an opportunity for lifestyle counseling [8], with PA counseling an excellent opportunity for advice and discussion between healthcare provider and individual patient [9].

A primary care setting provides an appropriate environment for PA counseling since it offers a first contact between primary care or family physicians and the majority of the population [10, 11]. PA assessment and advice, as part of routine healthcare delivery, is one of the best means by which to promote PA [12]. Primary care providers should encourage PA assessment at each clinic visit, as well as set PA goals and monitor PA behaviors [8]. A systematic review of 19 studies, conducted with primary care providers, indicated that “a lack of time” was the most cited barrier to PA counseling (14 out of 19 studies). The second most common barrier was “a lack of knowledge or training in PA counseling” (8 out of 19 studies) [13]. This finding was consistent with a recent systematic review that demonstrated that the lack of proper PA education was the most important barrier to PA promotion [14]. Hence, structured PA medical education and training is essential.

A recent systematic review focused on PA counseling in undergraduate medical school education [15]. The review assessed 10 programs from four countries, which is insufficient to determine the quality and effectiveness of those programs [15]. Moreover, a lack of organized education in PA counseling and lifestyle medicine during residency training is also a challenge [16]. Further, no assessment has systematically measured PA counseling of primary care residency programs. Hence, the purpose of this investigation was to systematically evaluate PA counseling in primary care, residency program training. The definition of primary care residency training varies across countries [17]. Herein, primary care residency training includes; family medicine training, general practitioner training, and any residency training in a primary care setting. This systematic review focused on postgraduate primary care trainees as the participants, and the training programs as the interventions for PA counseling. The findings provide a comprehensive understanding of the training for PA counseling during primary care residency. This understanding provides insights for policymakers, academics, and professional societies that will ensure the development of effective policies and curricula for PA for the next generation of primary care physicians and their patients.

## Methods

### Search methods

We conducted a systematic literature search of four databases: PubMed, Web of Science, Scopus, and The Cochrane Library. The search terms and Medical Subject Headings (MeSH) terms were the combination of “exercise”, “physical activity”, “counseling”, “prescribe”, “prescription”, “family medicine”, “family physician”, “family practice”, “general practitioner”, “primary care physician”, “curriculum”, “education”, “residency”, “resident”, “teaching”, and “training” (Details of search strategies are in the Additional file 1). The search strategy was intentionally broad because the definitions and terms related to physical activity, primary care physician, and residency training were inconsistent between relevant studies. The filter function was used to recruit studies published in English from the years 2000 to September 2017.

### Study selection

A total of 453 articles were identified from the database search. After removal of duplicates, 382 potential articles remained. Two investigators (AW and TT) independently screened titles and abstracts using an online systematic review toolkit, Covidence (https://www.covidence.org/). Relevant studies were selected based on search queries for medical education, which consisted of three components; participants, educational aspects, and outcomes [18]. Table 1 shows a summary of inclusion and exclusion criteria for study selection. Any differences in title and abstract screening were resolved through discussion by the two reviewers (AW and TT). A third reviewer (ST) participated in conflict resolution between the two reviewers.

**Table 1:** Inclusion and exclusion criteria for study selection

Component	Inclusion criteria	Exclusion criteria
Study design	Any observational or experimental design (including both quantitative and qualitative studies)	Not applicable
Type of literature and specific details	Primary studies (e.g. research or original articles) published in English language only	Secondary studies (e.g. review articles – systematic or narrative reviews)
Participants	Postgraduate primary care physician trainees of any year from any institution or comparable populations in their training: - Primary care - Family medicine - Family practice - Family physician - General practitioner	Undergraduate medical students and other specialty training
Educational aspects	Educational programs or training that address PA counseling or relevant training: - PA prescription - Exercise counseling - Exercise prescription	Lifestyle counseling programs that did not include PA or exercise components
Outcomes	Studies that provided at least one outcome relating to PA counseling training (e.g. program structures, learner’s outcomes, or patient’s outcomes)	Studies that provided insufficient data related to PA counseling training
		Studies with outcomes that did not link to educational programs

### Data synthesis and analysis

Two researchers (AW and TT) independently performed data extraction for each identified study. The extraction form used was developed according to the Centre for Reviews and Dissemination (CRD) guidance for undertaking reviews in health care, which included general information about the review, study characteristics, participant characteristics, intervention, setting, and outcome data [19]. The main study results (both descriptive and analytical) regarding PA counseling in primary care physician training were summarized as the outcomes of this systematic review. Disagreements among analysts were resolved by consensus (AW, TT, and ST). The Mixed Methods Appraisal Tool (MMAT) was used to appraise the methodological quality of each study. Scores varied from 25% (one criterion met) to 100% (all criteria met) [20–22].

## Results

**Fig. 1:**
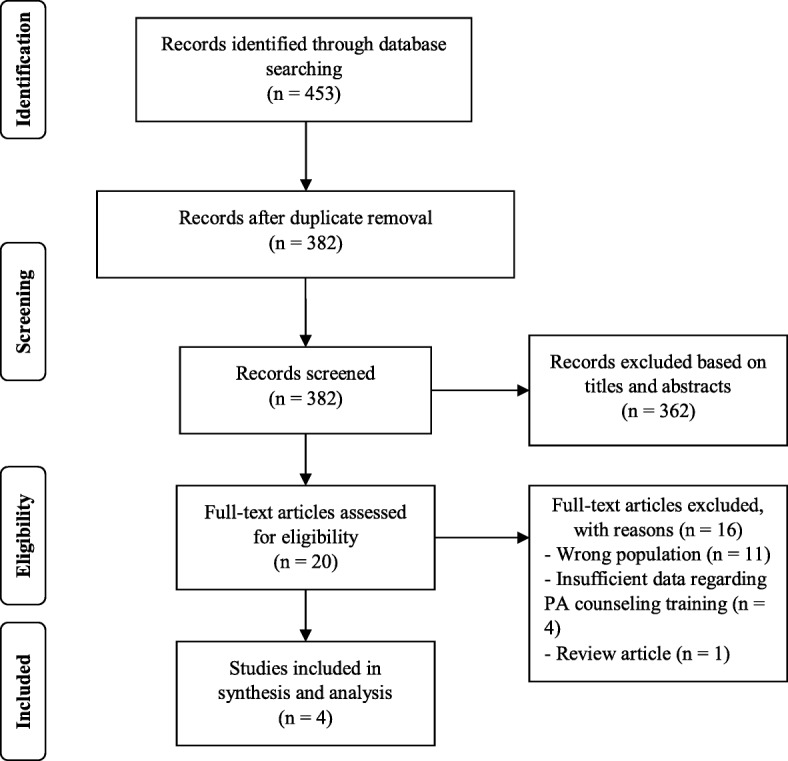
Flow diagram for study selection

Based on the initial review, 362 articles were excluded, leaving 20 eligible articles for full text review. Two reviewers (AW and TT) separately reviewed the selected full texts (*n* = 20). An additional 16 articles were excluded after the full text review, leaving a total of four articles for data synthesis and analysis (Fig. 1). Four primary studies from the USA [23–25] and Israel [26] were included in the review. Two studies provided counseling programs regarding obesity, nutrition, and physical activity (ONPA) [23, 25]. One study emphasized obesity counseling, including PA [24]. The other focused on lifestyle medicine [26]. Table 2 presents a summary of the included studies.

**Table 2:** Content summary for the four included studies

Authors and year	Methodological quality^*^	Study design	Participant and setting	Educational programs/training	Outcomes
Antognoli et al. [23] 2017	75%	Mixed methods study - Document review and audit: program-level demographic and curricular information (*n* = 25) - Cross-sectional survey: resident’s knowledge, attitudes, self-efficacy, and perceived professional norms (*n* = 219) - In-depth interviews: program member’s perceptions regarding ONPA counseling in primary care residency education (*n* = 84)	Primary care residency programs in the state of Ohio, including family medicine (FM), internal medicine (IM), OB/GYN residents and faculty members	ONPA training	Program structure: ONPA-related training opportunities
					Physician outcomes: knowledge, attitudes, self-efficacy, and perceived professional norms
					Patient outcomes: N/A
Malatskey et al. [26] 2017	75%	Pre- and post-course survey (*n* = 91) to investigate resident’s knowledge, attitudes, self-efficacy, personal health survey, and teaching feedback	Israeli family medicine residents at Tel Aviv University and “HaEmek” medical center	A 20-h lifestyle medicine course including 4 h of PA basic concepts and prescription	Program structure: topics and allocation of academic hours
					Physician outcomes: knowledge, attitudes, self-efficacy, personal health, and teaching feedback
					Patient outcomes: N/A
Smith et al. [25] 2015	75%	Cross-sectional survey to investigate associations among ONPA counseling, demographic, and training program characteristics (*n* = 219)	Primary care residents (FM, IM, and OB/GYN) across Ohio	ONPA counseling	Program structure: N/A
					Physician outcomes: associations among ONPA counseling scores (knowledge, attitudes, self-efficacy, and professional norms) and training program characteristics
					Patient outcomes: N/A
Jay et al. [24] 2010	75%	Non-randomized controlled trial to explore rate of counseling and quality of counseling from patient interviews - Intervention group (curriculum): 12 residents with 82 patients interviewed - Control group (no curriculum): 11 residents with 76 patients interviewed	Primary care residents in a residency program at Gouverneur Healthcare Services, part of the New York City Health and Hospitals Corporation	A 5-h obesity counseling curriculum based on the 5As (assess, advise, agree, assist, arrange) using case-study, role play, standardized patients, faculty-facilitated videotape review, and counseling skills (behavioral assessment, goal setting, and motivational interviewing)	Program structure: designed obesity counseling curriculum for the intervention group
					Physician outcomes: obesity counseling performance
					Patient outcomes: number of patients counseled about obesity

*FM* Family medicine, *IM* Internal medicine, *N/A* Not available, *OB/GYN* Obstetrics/gynecology, *ONPA* Obesity, nutrition, and physical activity, *PA* Physical activity

*Please see the Additional file 2

All four studies were appraised for the methodological quality by using the MMAT (scoring 25 to 100%). The study of Antognoli et al. was a mixed methods study and was scored at 75% [23]. Three quantitative studies were rated at 75% by using the criteria for descriptive study [25, 26] and non-randomized trial [24]. Table 2 shows the methodological quality of each study. The full results of quality assessment were shown in the Additional file 2.

### Program structure

All included primary care residency programs described PA counseling or prescription as part of ONPA, obesity, or lifestyle medicine training [23–26]. Each provided different program structure [23–26]. According to a study of Antognoli et al., the survey of the USA primary care residency programs found that only four of the 25 eligible residency programs (17%) provided use and implementation of ONPA guidelines; 10 programs (42%) offered health behavior counseling techniques; six programs (24%) afforded ONPA-related electives; and six programs (24%) delivered ONPA-related fellowships [23]. The programs contributed to a small proportion of ONPA-related topics (2.8 ± 4.9 h per year) compared to the didactic teaching (225 ± 96 h per year) [23]. By interview of primary care residency program members, improved approaches for ONPA counseling training were; combinational training in counseling techniques; opportunities for role-playing, observation, feedback, and increased allied-health professional engagement [23].

In a pre- and post-interventional study, conducted with Israeli family medicine residents, training included: 20 h of lifestyle medicine training; 4 h of PA basic concepts and PA prescription; and 2 h of stress, resilience, and yoga lessons [26]. Residents reported a high level of satisfaction with the following course domains: contribution to professional training (4.42/5), professional level of teachers (4.46/5), ability to actively participate (4.4/5), and scientific level of the course materials (4.34/5) [26].

One study provided 5 h of an obesity counseling curriculum based on the 5As (assess, advise, agree, assist, arrange) that used multiple active-instructional methods including; case-study, role play, standardized patients, faculty-facilitated videotape review, and counseling skills [24].

### Physician outcomes

Three studies presented knowledge, attitudes, and self-efficacy as physician outcomes [23, 25, 26]. Antognoli et al. found that with formal teaching of ONPA-related didactics and ONPA guidelines, as well as of health-behavior change, counseling techniques were significantly associated with greater ONPA knowledge among primary care residents [23]. Paradoxically, that study showed that ONPA training opportunities were associated with poorer attitudes and perceived professional norms [23]. A similar trend was found in a study by Melatsky et al., wherein 20 h of lifestyle medicine training improved residents’ knowledge and lifestyle medicine consultation self-efficacy, but did not improve attitudes for healthy lifestyle consultation [26]. Participation in elective rotations that focused on ONPA were positively associated with attitudes, self-efficacy, and professional norms [23, 25]. In contrast, offering ONPA-related fellowships had no association with knowledge, attitudes, self-efficacy, or professional norms [23].

One study focused on counseling skills using the 5As approach [24]. There were some differences in counseling between residents enrolled in the intervention (curriculum) or in the control (no curriculum) group, but those differences were not statistically significant [24]. The positive trends for the quality of counseling among residents in the intervention group were found; overall counseling (36.6% vs 31.2%, *p* = 0.21), advanced counseling (27.4% vs 35.9%, *p* = 0.10), “assess” (46.7% vs 38.2%, *p* = 0.15), “advise” (46.0% vs 36.2%, p = 0.10), “agree” (26.8% vs 18.7%, *p* = 0.20), and “assist” (22.4% vs 16.4%, *p* = 0.36) [24]. In contrast, residents in the intervention group had poorer performance in “arrange” compared with the control group (20.0% vs 21.3%, *p* = 0.74) [24].

In terms of health behaviors, the number of residents who reported regular PA (3–4 times/week for at least 30 min) did not increase significantly (36.8 to 42%, *p* = 0.52) after the lifestyle medicine course, although there was a significant change among overweight residents (12% vs 21%, *p* = 0.05) [26].

### Patient outcomes

Jay et al. compared the number of patients counseled about obesity by residents in the intervention (curriculum – 78 patients) and control (no curriculum – 74 patients) groups [24]. There was no significant difference in the proportion of patients who received counseling from their doctors between the intervention (73%, 57/78) and control (72%, 53/74) groups [24].

## Discussion

This systematic review assessed different PA counseling curricula for primary care residency training. All the included studies presented PA counseling training as part of training for obesity and healthy lifestyle counseling. The training provided for better knowledge among primary care residents but may not provide for better attitudes and self-efficacy toward PA counseling. Elective rotations provided obesity, nutrition, and PA counseling training that possibly improved positive attitudes, self-efficacy, and professional norms for the practice of lifestyle advice. However, brief training programs in obesity counseling did not necessarily improve quality nor increase the rate of counseling [24].

The included studies did not only focus on PA counseling but also on obesity and lifestyle counseling [23–26]. Lifestyle modification programs were holistic or comprised specific modules. The combination of nutrition and PA [27], behavior change [28], and preventive counseling [29] were considered holistic modules, while a number of training programs focused specifically on PA [30–33]. In undergraduate medical training, PA was always incorporated into the curriculum with other components, e.g., nutrition with health behavior change, or with healthy aging, or with disease prevention [15]. PA training programs for counseling should integrate cognitive and behavioral components to ensure greater motivation as well as greater patient support (more than just advice and education) [34]. It is important to note that compared to undergraduate medical training, residency training requires synthesis and evaluation of counseling based on experience and expertise [35].

Insufficient PA knowledge was a challenge for family medicine residents [36]. Structured PA curricula were effective at improving knowledge among primary care residents [23, 24, 26], and health professionals [30, 37]. However, training programs did not guarantee attitude improvement or the self-efficacy of primary care residents. This may be due to the perception of barriers by trainees (e.g. lack of time, difficulty in changing patient behaviors, and insufficient PA counseling protocols) and the complexity of PA counseling [13, 23, 26, 38]. A number of studies evaluated PA counseling quality through the 5As framework, which revealed that brief educational courses (3–5 h) did not significantly improve the quality of counseling [24, 39]. This finding demonstrates a need for effective educational PA counseling programs that are more than knowledge-oriented.

A strength of this systematic review was the recruitment of primary studies from both medical (PubMed and The Cochrane Library) and multi-disciplinary (Web of Science and Scopus) databases, with a search strategy focused on primary care residency training and the clarification of specific outcomes for the targeted population. The included studies scored 75% for methodological quality, reflecting high internal validity. However, there were limitations to this investigation. First, the definition of primary care residency training programs varied across countries. Second, the small number of included studies may not provide an adequate number to fully evaluate program structures, physician, and patient outcomes. The small number of available studies may be due to a lack of PA counseling in primary care residency training, or the fact that such training may be taught in lifestyle medicine or health promotion. Third, the included studies were conducted in the USA or Israel, which may limit the generalization of findings.

## Conclusion

This systematic review demonstrates a lack of evidence due to a small number of included studies. The heterogeneous outcomes from the minimal programs are needed to carefully interpret. However, this review sheds light on the importance of training in PA counseling in primary care residency programs. A lack of knowledge and training are considered barriers to PA counseling. This reflects a gap in primary care residency training. Elective rotations, where residents voluntarily choose their subject, may provide the appropriate training period for PA counseling. The development of a PA counseling curriculum should focus on an approach that improves attitudes and the self-efficacy of primary care residents, with an emphasis on patient outcomes. Policymakers and academics should implement PA counseling as an essential competency for primary care physicians. Further studies should highlight program delivery methods, changes in resident level outcomes (e.g. self-efficacy and PA behaviors), and approaches to address PA counseling in primary care residency training.

## Additional files

Additional file 1: Search strategy. (DOCX 17 kb)

Additional file 2: Results of quality assessment using Mixed Methods Appraisal Tool (MMAT). (DOCX 15 kb)

## Abbreviations

ONPA: Obesity, nutrition, and physical activity
PA: Physical activity
