# Supplementary material for: Physical activity counseling in primary care and family medicine residency training: a systematic review
Source: BMC Med Educ. 2018 Jul 3;18:159. doi: 10.1186/s12909-018-1268-1 (PMC6029015; doi:10.1186/s12909-018-1268-1)
Supplement: Supplementary file 1 — Search strategy. (DOCX 17 kb) [file 12909_2018_1268_MOESM1_ESM.docx]

**Additional File 1: Search strategy**

**1. PubMed (n = 221)**

| **Number** | **Search Strategy** |
| --- | --- |
| # 16 | Search (#5) AND #14 Sort by: Relevance Filters: Publication date from 2000/01/01 to 2017/09/12 |
| # 15 | #5 OR #14 |
| # 14 | #10 OR #13 |
| # 13 | #11 OR #12 |
| # 12 | curriculum OR education OR teaching OR training |
| # 11 | residency [MH] |
| # 10 | #6 OR #7 OR #8 OR #9 |
| # 9 | primary care physician [MH] |
| # 8 | general practitioner [MH] |
| # 7 | "family medicine" OR "family physician" OR "family physicians" |
| # 6 | Family medicine [MH] |
| # 5 | #3 OR #4 |
| # 4 | counseling OR prescription |
| # 3 | #1 OR #2 |
| # 2 | "Exercises" OR "Physical Activity" OR "Activities, Physical" OR "Activity, Physical" OR "Physical Activities" OR "Exercise, Physical" OR "Exercises, Physical" OR "Physical Exercise" OR "Physical Exercises" OR "Acute Exercise" OR "Acute Exercises" OR "Exercise, Acute" OR "Exercises, Acute" OR "Exercise, Isometric" OR "Exercises, Isometric" OR "Isometric Exercises" OR "Isometric Exercise" OR "Exercise, Aerobic" OR "Aerobic Exercise" OR "Aerobic Exercises" OR "Exercises, Aerobic" OR "Exercise Training" OR "Exercise Trainings" OR "Training, Exercise" OR "Trainings, Exercise" |
| # 1 | Physical activity [MH] |

**2. Web of Science (n=197)**

| **Number** | **Search Strategy** |
| --- | --- |
| # 13 | #3 OR #12 |
| # 12 | #8 OR #11 |
| # 11 | #9 OR #10 |
| # 10 | curriculum OR education OR teaching OR training |
| # 9 | residency |
| # 8 | #4 OR #5 OR #6 OR #7 |
| # 7 | Family medicine |
| # 6 | primary care physician |
| # 5 | general practitioner |
| # 4 | "family medicine" OR "family physician" OR "family physicians" |
| # 3 | (#1 AND #2) AND ENGLISH |
| # 2 | counseling OR prescription |
| # 1 | "Exercises" OR "Physical Activity" OR "Activities, Physical" OR "Activity, Physical" OR "Physical Activities" OR "Exercise, Physical" OR "Exercises, Physical" OR "Physical Exercise" OR "Physical Exercises" OR "Acute Exercise" OR "Acute Exercises" OR "Exercise, Acute" OR "Exercises, Acute" OR "Exercise, Isometric" OR "Exercises, Isometric" OR "Isometric Exercises" OR "Isometric Exercise" OR "Exercise, Aerobic" OR "Aerobic Exercise" OR "Aerobic Exercises" OR "Exercises, Aerobic" OR "Exercise Training" OR "Exercise Trainings" OR "Training, Exercise" OR "Trainings, Exercise" |

**3. Scopus (n=20)**

| **Number** | **Search Strategy** |
| --- | --- |
| # 14 | #5 and #13 Publication Year from 2000 to 2017 |
| # 13 | #9 and #12 |
| # 12 | #10 or #11 |
| # 11 | curriculum or education or teaching or training |
| # 10 | MeSH descriptor: [Internship and Residency] explode all trees |
| # 9 | #6 or #7 or #8 |
| # 8 | MeSH descriptor: [Physicians, Primary Care] explode all trees |
| # 7 | MeSH descriptor: [General Practitioners] explode all trees |
| # 6 | "family medicine" or "family physician" or "family physicians" |
| # 5 | #3 and #4 |
| # 4 | counseling or prescription |
| # 3 | #1 and #2 |
| # 2 | "Exercises" or "Physical Activity" or "Activities, Physical" or "Activity, Physical" or "Physical Activities" or "Exercise, Physical" or "Exercises, Physical" or "Physical Exercise" or "Physical Exercises" or "Acute Exercise" or "Acute Exercises" or "Exercise, Acute" or "Exercises, Acute" or "Exercise, Isometric" or "Exercises, Isometric" or "Isometric Exercises" or "Isometric Exercise" or "Exercise, Aerobic" or "Aerobic Exercise" or "Aerobic Exercises" or "Exercises, Aerobic" or "Exercise Training" or "Exercise Trainings" or "Training, Exercise" or "Trainings, Exercise" |
| # 1 | MeSH descriptor: [Exercise] explode all trees |

**4. Cochrane Library (n =15)**

| **Number** | **Search Strategy** |
| --- | --- |
| # 14 | #5 and #13 Publication Year from 2000 to 2017 |
| # 13 | #9 and #12 |
| # 12 | #10 or #11 |
| # 11 | curriculum or education or teaching or training |
| # 10 | MeSH descriptor: [Internship and Residency] explode all trees |
| # 9 | #6 or #7 or #8 |
| # 8 | MeSH descriptor: [Physicians, Primary Care] explode all trees |
| # 7 | MeSH descriptor: [General Practitioners] explode all trees |
| # 6 | "family medicine" or "family physician" or "family physicians" |
| # 5 | #3 and #4 |
| # 4 | counseling or prescription |
| # 3 | #1 and #2 |
| # 2 | "Exercises" or "Physical Activity" or "Activities, Physical" or "Activity, Physical" or "Physical Activities" or "Exercise, Physical" or "Exercises, Physical" or "Physical Exercise" or "Physical Exercises" or "Acute Exercise" or "Acute Exercises" or "Exercise, Acute" or "Exercises, Acute" or "Exercise, Isometric" or "Exercises, Isometric" or "Isometric Exercises" or "Isometric Exercise" or "Exercise, Aerobic" or "Aerobic Exercise" or "Aerobic Exercises" or "Exercises, Aerobic" or "Exercise Training" or "Exercise Trainings" or "Training, Exercise" or "Trainings, Exercise" |
| # 1 | MeSH descriptor: [Exercise] explode all trees |
