# Supplementary material for: Physical activity counseling in primary care and family medicine residency training: a systematic review
Source: BMC Med Educ. 2018 Jul 3;18:159. doi: 10.1186/s12909-018-1268-1 (PMC6029015; doi:10.1186/s12909-018-1268-1)
Supplement: Supplementary file 2 — Results of quality assessment using Mixed Methods Appraisal Tool (MMAT). (DOCX 15 kb) [file 12909_2018_1268_MOESM2_ESM.docx]

**Additional File 2: Results of quality assessment using Mixed Methods Appraisal Tool (MMAT)**

1. Title: Primary care resident training for obesity, nutrition, and physical activity counseling: a mixed-methods study

Authors: Antognoli et al.

Type of study: Mixed methods

Score: 75%

| **Study components** | **Methodological quality criteria** | **Response** | | | |
| --- | --- | --- | --- | --- | --- |
|  |  | **Yes** | **No** | **Can’t tell** | **Comments** |
| Screening questions | Are there clear qualitative and quantitative research questions (or objectives*), or a clear mixed methods question (or objective*)? | **🗸** |  |  | 2/2 |
|  | Do the collected data allow address the research question (objective)? E.g., consider whether the follow-up period is long enough for the outcome to occur (for longitudinal studies or study components). | **🗸** |  |  |  |
| Qualitative | Are the sources of qualitative data (archives, documents, informants, observations) relevant to address the research question  (objective)? | **🗸** |  |  | 4/4 |
|  | Is the process for analyzing qualitative data relevant to address the research question (objective)? | **🗸** |  |  |  |
|  | Is appropriate consideration given to how findings relate to the context, e.g., the setting, in which the data were collected? | **🗸** |  |  |  |
|  | Is appropriate consideration given to how findings relate to researchers’ influence, e.g., through their interactions with participants? | **🗸** |  |  |  |
| Quantitative  descriptive | Is the sampling strategy relevant to address the quantitative research question (quantitative aspect of the mixed methods question)? | **🗸** |  |  | 3/4 |
|  | Is the sample representative of the population understudy? |  |  | **🗸** |  |
|  | Are measurements appropriate (clear origin, or validity known, or standard instrument)? | **🗸** |  |  |  |
|  | Is there an acceptable response rate (60% or above)? | **🗸** |  |  |  |
| Mixed methods | Is the mixed methods research design relevant to address the qualitative and quantitative research questions (or objectives), or the qualitative and quantitative aspects of the mixed methods question (or objective)? | **🗸** |  |  | 2/3 |
|  | Is the integration of qualitative and quantitative data (or results*) relevant to address the research question (objective)? | **🗸** |  |  |  |
|  | Is appropriate consideration given to the limitations associated with this integration, e.g., the divergence of qualitative and quantitative data (or results*) in a triangulation design? |  |  | **🗸** |  |

2. Title: Lifestyle medicine course for family medicine residents: preliminary assessment of the impact on knowledge, attitudes, self-efficacy and personal health

Authors: Malatskey et al.

Type of study: Quantitative descriptive

Score: 75%

| **Study components** | **Methodological quality criteria** | **Response** | | | |
| --- | --- | --- | --- | --- | --- |
|  |  | **Yes** | **No** | **Can’t tell** | **Comments** |
| Screening questions | Are there clear qualitative and quantitative research questions (or objectives*), or a clear mixed methods question (or objective*)? | **🗸** |  |  | 2/2 |
|  | Do the collected data allow address the research question (objective)? E.g., consider whether the follow-up period is long enough for the outcome to occur (for longitudinal studies or study components). | **🗸** |  |  |  |
| Quantitative  descriptive | Is the sampling strategy relevant to address the quantitative research question (quantitative aspect of the mixed methods question)? | **🗸** |  |  | 3/4 |
|  | Is the sample representative of the population understudy? |  | **🗸** |  |  |
|  | Are measurements appropriate (clear origin, or validity known, or standard instrument)? | **🗸** |  |  |  |
|  | Is there an acceptable response rate (60% or above)? | **🗸** |  |  |  |

3. Title: Primary care residents’ knowledge, attitudes, self-efficacy, and perceived professional norms regarding obesity, nutrition, and physical activity counseling

Authors: Smith et al.

Type of study: Quantitative descriptive

Score: 75%

| **Study components** | **Methodological quality criteria** | **Response** | | | |
| --- | --- | --- | --- | --- | --- |
|  |  | **Yes** | **No** | **Can’t tell** | **Comments** |
| Screening questions | Are there clear qualitative and quantitative research questions (or objectives*), or a clear mixed methods question (or objective*)? | **🗸** |  |  | 2/2 |
|  | Do the collected data allow address the research question (objective)? E.g., consider whether the follow-up period is long enough for the outcome to occur (for longitudinal studies or study components). | **🗸** |  |  |  |
| Quantitative  descriptive | Is the sampling strategy relevant to address the quantitative research question (quantitative aspect of the mixed methods question)? | **🗸** |  |  | 3/4 |
|  | Is the sample representative of the population understudy? |  |  | **🗸** |  |
|  | Are measurements appropriate (clear origin, or validity known, or standard instrument)? | **🗸** |  |  |  |
|  | Is there an acceptable response rate (60% or above)? | **🗸** |  |  |  |

4. Title: From the patient’s perspective: the impact of training on resident physician’s obesity counseling

Authors: Jay et al.

Type of study: Quantitative non-randomized

Score: 75%

| **Study components** | **Methodological quality criteria** | **Response** | | | |
| --- | --- | --- | --- | --- | --- |
|  |  | **Yes** | **No** | **Can’t tell** | **Comments** |
| Screening questions | Are there clear qualitative and quantitative research questions (or objectives*), or a clear mixed methods question (or objective*)? | **🗸** |  |  | 2/2 |
|  | Do the collected data allow address the research question (objective)? E.g., consider whether the follow-up period is long enough for the outcome to occur (for longitudinal studies or study components). | **🗸** |  |  |  |
| Quantitative non-randomized | Are participants (organizations) recruited in a way that minimizes selection bias? | **🗸** |  |  | 3/4 |
|  | Are measurements appropriate (clear origin, or validity known, or standard instrument; and absence of contamination between groups when appropriate) regarding the exposure/intervention and outcomes? |  |  | **🗸** |  |
|  | In the groups being compared (exposed vs. non-exposed; with intervention vs. without; cases vs. controls), are the participants comparable, or do researchers take into account (control for) the difference between these groups? | **🗸** |  |  |  |
|  | Are there complete outcome data (80% or above), and, when applicable, an acceptable response rate (60% or above), or an acceptable follow-up rate for cohort studies (depending on the duration of follow-up)? | **🗸** |  |  |  |

Ref: http://mixedmethodsappraisaltoolpublic.pbworks.com/w/file/fetch/84371689/MMAT%202011%20criteria%20and%20tutorial%202011-06-29updated2014.08.21.pdf
